# A preoperative model based on gadobenate-enhanced MRI for predicting microvascular invasion in hepatocellular carcinomas (≤ 5 cm)

**DOI:** 10.3389/fonc.2022.992301

**Published:** 2022-08-30

**Authors:** Sisi Zhang, Lei Huo, Juan Zhang, Yayuan Feng, Yiping Liu, Yuxian Wu, Ningyang Jia, Wanmin Liu

**Affiliations:** ^1^ Department of Radiology, Shanghai Eastern Hepatobiliary Surgery Hospital, The Third Affiliated Hospital of Naval Medical University, Shanghai, China; ^2^ Department of Radiology, Tongji Hospital, School of Medicine, Tongji University, Shanghai, China

**Keywords:** gadobenate dimeglumine, hepatocellular carcinoma, microvascular invasion, magnetic resonance imaging, nomogram

## Abstract

**Purpose:**

The present study aimed to develop and validate a preoperative model based on gadobenate-enhanced magnetic resonance imaging (MRI) for predicting microvascular invasion (MVI) in patients with hepatocellular carcinoma (HCC) size of ≤5 cm. In order to provide preoperative guidance for clinicians to optimize treatment options.

**Methods:**

164 patients with pathologically confirmed HCC and preoperative gadobenate-enhanced MRI from July 2016 to December 2020 were retrospectively included. Univariate and multivariate logistic regression (forward LR) analyses were used to determine the predictors of MVI and the model was established. Four-fold cross validation was used to verify the model, which was visualized by nomograms. The predictive performance of the model was evaluated based on discrimination, calibration, and clinical utility.

**Results:**

Elevated alpha-fetoprotein (HR 1.849, 95% CI: 1.193, 2.867, P=0.006), atypical enhancement pattern (HR 3.441, 95% CI: 1.523, 7.772, P=0.003), peritumoral hypointensity on HBP (HR 7.822, 95% CI: 3.317, 18.445, P<0.001), and HBP hypointensity (HR 3.258, 95% CI: 1.381, 7.687, P=0.007) were independent risk factors to MVI and constituted the HBP model. The mean area under the curve (AUC), sensitivity, specificity, and accuracy values for the HBP model were as follows: 0.830 (95% CI: 0.784, 0.876), 0.71, 0.78, 0.81 in training set; 0.826 (95% CI:0.765, 0.887), 0.8, 0.7, 0.79 in test set. The decision curve analysis (DCA) curve showed that the HBP model achieved great clinical benefits.

**Conclusion:**

In conclusion, the HBP imaging features of Gd-BOPTA-enhanced MRI play an important role in predicting MVI for HCC. A preoperative model, mainly based on HBP imaging features of gadobenate-enhanced MRI, was able to excellently predict the MVI for HCC size of ≤5cm. The model may help clinicians preoperatively assess the risk of MVI in HCC patients so as to guide clinicians to optimize treatment options.

## Introduction

Hepatocellular carcinoma (HCC) is the sixth most common neoplasm and the third leading cause of death from cancer in the world (1, 2). While numerous treatment strategies have been developed, HCC patients remain at a high risk of tumor recurrence (3, 4). Microvascular invasion (MVI) is an important prognostic factor in patients with HCC and is associated with early recurrence and poor survival (5, 6). However, MVI diagnosis currently requires histopathological analysis of the surgical specimens, which can only be performed postoperatively (7). Previous study demonstrated that enlarged surgical margin (usually over 1cm) could reduce postoperative tumor recurrence rates in MVI-positive patients with HCC (8). And postoperative adjuvant transarterial chemoembolization could improve the overall survival and disease-free survival for patients who have HCC with MVI (9). Therefore, the preoperative prediction of MVI in patients with HCC is necessary for clinicians to optimize treatment options and improve long-term survival (10). Some evidence shows that tumor size is correlated to the incidence of MVI (11). This implies tumor size may be a potential confounding factor in predicting MVI. Meanwhile, early diagnosis of MVI in patients with HCC, especially patients with HCC size of ≤5 cm, will help clinicians choose more appropriate therapeutic regimens to improve prognosis.

Gadobenate dimeglumine (Gd-BOPTA) is a hepatobiliary-specific agent, it can be selectively taken up into function hepatocytes by the specific organic anion transporting polypeptides (OATP1B1, OATP1B3) located on the surface of hepatocytes (12–14). In addition to dynamic contrast-enhanced MRI of the liver, this agent may also assist with specific imaging in the hepatobiliary phase (HBP) within 40–120 min after injection (15). Compared to gadoxetate disodium-enhanced MRI, Gd-BOPTA-enhanced MRI have a true delayed phase (DP), instead of a transitional phase (TP) (15). Previous studies have reported some models for predicting MVI using gadoxetate disodium-enhanced MRI (16, 17), but no previous literature has reported the use of gadolinate-enhanced MRI to build a model to predict MVI. The present investigation used the true DP and HBP imaging features based on gadobenate-enhanced MRI to preoperatively predict MVI in patients with HCC.

Accordingly, the present study aims to develop and validate a preoperative model based on gadobenate-enhanced MRI for predicting MVI in patients with HCC size of ≤5 cm. In order to provide preoperative guidance for clinicians to optimize treatment options.

## Materials and methods

### Patients

The present study was approved by the ethics committee of the Eastern Hepatobiliary Surgery Hospital, the Third Affiliated Hospital of Shanghai Naval Military Medical University, China. The requirement for written informed consent was waived.

**Figure 1:**
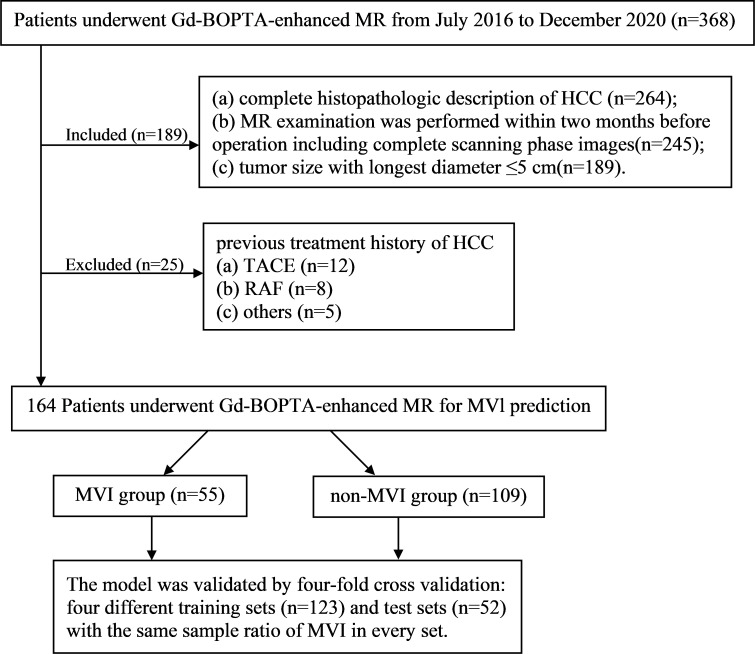
The workflow of patient selection for this study.

Between July 2016 and December 2020, a total of 164 pathologically confirmed HCC patients (138 males and 26 females; 55.13 ± 10.52 years) after preoperative Gd-BOPTA-enhanced MRI met the following inclusion criteria (**Figure 1**): (a) tumor size with the longest diameter of ≤5 cm; (b) complete histopathologic HCC description; (c) Gd-BOPTA-enhanced MR examination was performed within two months before the operation, including complete scanning phase images (arterial phase, portal phase, DP, and HBP); and (d) no previous treatment history of HCC, such as liver transplantation, transarterial chemoembolization, radiofrequency ablation.

### Laboratory examinations and histopathology

Preoperative laboratory indexes (**Table 1**) comprised protein induced by vitamin K absence/antagonist-II (PIVKA-II), serum alpha-fetoprotein (AFP), AFP-L3, carbohydrate antigen 19-9, carcinoembryonic antigen, hepatitis B virus (HBV), HBV-DNA loads, anti-hepatitis C virus, alanine aminotransferase, aspartate aminotransferase, total bilirubin, direct bilirubin, cholinesterase (CHE), r-glutamyltransferase, α-L-fucosidase, total protein, albumin (ALB), total cholesterol, prothrombin time, activated partial thromboplastin time.

**Table 1:** Clinicoradiological characteristics for predicting MVI.

Characteristic	Total (n = 164)	Non-MVI (n = 109)	MVI (n = 55)	P value
**Clinical features**				
Age (y)	55.13 ± 10.52	54.29 ± 10.61	56.78 ± 10.24	0.158
Sex				
Male	138 (84.1%)	89 (81.7%)	49 (89.1%)	0.263
Female	26 (15.9%)	20 (18.3%)	6 (10.9%)	
Liver disease				
HBV	149 (90.9%)	97 (89.0%)	52 (94.5%)	0.390
None	10 (6.1%)	8 (7.3%)	2 (3.6%)	
Other	5 (3.0%)	4 (3.7%)	1 (1.8%)	
Child-Pugh				
A	157 (95.7%)	102 (93.6%)	55 (100%)	0.102
B	7 (4.3%)	7 (6.4%)	0 (0.0%)	
HBV/C-DNA (IU/ml)				
≤50	80 (48.8%)	54 (49.5%)	26 (47.3%)	0.808
50-10^3	25 (15.2%)	17 (15.6%)	8 (14.5%)	
10^3-10^5	27 (16.5%)	19 (17.4%)	8 (14.5%)	
≥10^5	32 (19.5%)	19 (17.4%)	13 (23.6%)	
AFP-L3				
Negative	110 (67.1%)	77 (70.6%)	33 (60.0%)	0.171
Positive	54 (32.9%)	32 (29.4%)	22 (40.0%)	
AFP (ng/L)	29.4 (4.8,185.7)	15.1 (3.3,118.8)	60.3 (12.3,341.1)	0.023
AFP_lg10 (ng/L)	1.46 (0.68,2.27)	1.18 (0.51,2.07)	1.78 (1.09,2.53)	0.002
PIVKA-II (mAU/mL)	56 (28,257.8)	56 (24,239.5)	94 (37,410)	0.432
PIVKA-II_lg10	1.85 (1.39,2.57)	1.73 (1.36.2.47)	1.98 (1.45,2.61)	0.467
CA199 (U/mL)	16.3 (7.8,30.2)	17.1 (7.9,28.4)	16.3 (7.7,33.0)	0.713
CEA (ng/mL)	2.4 (1.6,3.2)	2.3 (1.6,3.2)	2.5 (1.6,3.4)	0.529
ALT (U/L)	28 (19.3,40.8)	28 (19,39)	30 (22,48)	0.249
AST (U/L)	25.5 (20.0,36.8)	26 (19,35)	25 (21,37)	0.290
TP (g/L)	68.4 (65.1,73.3)	68.5 (65.6,73.2)	68.3 (64.5,73.7)	0.628
ALB (g/L)	42.8 (39.9,45.7)	42.7 (39.6,45.6)	43.2 (40.5,46.0)	0.058
TBIL (μmol/L)	14.5 (12.0,18.6)	15.6 (12.2,18.6)	13.4 (11.6,18.6)	0.147
DBIL (μmol/L)	5.4 (4.3,7.2)	5.6 (4.4,7.2)	4.9 (4.1,6.9)	0.254
CHE (U/L)	7378 (5966,8258)	7153 (5339,8226)	7487 (6715,8297)	0.003
GGT (U/L)	43 (29,84)	44 (28,80)	40 (30,86)	0.574
AFU (U/L)	23 (19,29)	23 (19,28)	24 (18,32)	0.653
PT (S)	12 (11.4-12.6)	12.1 (11.4-12.9)	12.0 (11.3,12.5)	0.259
CHOL (mmol/L)	3.94 (3.41-4.38)	3.96 (3.42,4.45)	3.81 (3.39,4.34)	0.580
**Pathologic factors**				
Edmondson-Steiner grade				
I-II	24 (14.7%)	18 (16.5%)	6 (11.1%)	0.483
III-IV	139 (85.3%)	91 (83.5%)	48 (88.9%)	
Microscopic cirrhosis				
Absent	93 (58.1%)	61 (57.0%)	32 (60.4%)	0.735
Present	67 (41.9%)	46 (43.0%)	21 (39.6%)	
**MRI features**				
Tumor number				
Single	148 (90.2%)	102 (93.6%)	46 (83.6%)	0.128
Multiple	16 (9.8%)	7 (6.4%)	9 (16.4%)	
MRI Tumor diameter (cm)	2.7 (2.0,3.7)	2.6 (2.1,3.6)	2.9 (1.9,4.0)	0.559
Shape				
Regular	111 (67.7%)	77 (70.6%)	34 (61.8%)	0.290
Irregular	53 (32.3%)	32 (29.4%)	21 (38.2%)	
Margin				
Smooth	88 (53.7%)	65 (59.6%)	23 (41.8%)	0.032
Non-smooth	76 (46.3%)	44 (40.4%)	32 (58.2%)	
Radiological capsule				
Present	127 (77.4%)	90 (82.6%)	37 (67.3%)	0.029
Absent	37 (22.6%)	19 (17.4%)	18 (32.7%)	
Radiological capsule enhancement				
Complete	64 (39.0%)	52 (47.7%)	12 (21.8%)	0.002
Incomplete/Absent	100 (61.0%)	57 (52.3%)	43 (78.2%)	
Rim APHE				
Absent	123 (75.0%)	90 (82.6%)	33 (60.0%)	0.002
Present	41 (25.0%)	19 (17.4%)	22 (40.0%)	
Non-peripheral washout				
Present	105 (64.0%)	80 (73.4%)	25 (45.5%)	0.001
Absent	59 (36.0%)	29 (26.6%)	30 (54.5%)	
Enhancement pattern				
Typical	106 (64.6%)	81 (74.3%)	25 (45.5%)	<0.001
Atypical	58 (35.4%)	28 (25.7%)	30 (54.5%)	
Arterial peritumoral enhancement				
Absent	126 (76.8%)	90 (82.6%%)	36 (65.5%)	0.016
Present	38 (23.2%)	19 (17.4%)	19 (34.5%)	
Restricted diffusion				
Absent	10 (6.1%)	9 (8.3%)	1 (1.8%)	0.139
Present	154 (93.9)	100 (91.7)	54 (98.2)	
Hepatobiliary phase hypointensity				
Atypical	67 (40.9%)	55 (50.5%)	12 (21.8%)	0.001
Typical	97 (59.1%)	54 (49.5%)	43 (78.2%)	
Peritumoral hypointensity on HBP				
Absent	119 (72.6%)	95 (87.2%)	24 (43.6%)	<0.001
Present	45 (27.4%)	14 (12.8%)	31 (56.4%)	

MVI, microvascular invasion; HBV, hepatitis B virus; AFP, alpha-fetoprotein; PIVKA-II, protein induced by vitamin K absence or antagonist-II; CA199, carbohydrate antigen 19-9; CEA, carcinoembryonic antigen; ALT, alanine aminotransferase; AST, aspartate aminotransferase; TBIL, total bilirubin; DBIL, direct bilirubin; CHE, cholinesterase; ALB, albumin; GLOB, globulin; GGT, r-glutamyl transferase; AFU, a-fucosidase; PT, prothrombin time; CHOL, total cholesterol; APHE, arterial phase hyperenhancement; HBP, hepatobiliary phase.

The histopathological characteristics (tumor size, MVI status, and Edmondson-Steiner) were assessed by a consensus of two experienced pathologists. Until recently, MVI was defined as the presence of a tumor in a portal vein, hepatic vein, or large capsular vessel of the surrounding hepatic tissue lining the endothelium (17–20). The grades of MVI are classified as M0 (no MVI), M1 (invasion of microvessels up to five times at the peritumoral parenchyma within 1 cm of the tumor surface), and M2 (MVI at >5 sites or >1 cm away from the tumor surface) (11, 20). The cases were divided into the MVI (M1-2) (n=55) and non-MVI (M0) (n=109) groups.

### Gd-BOPTA-enhanced MR

MR images were acquired using a GE Optima MR360 1.5T (Optima MR360, GE Healthcare, USA) equipped with an eight-channel abdominal coil. Patients fasted for 4 h before the scan. Gd-BOPTA (MultiHance, Bracco) with a total dose of 0.1 mmoL/kg was injected into the median cubitus vein at a rate of 2.0 mL/s with a high-pressure syringe, followed by washing with 20 mL of normal saline. The arterial phase (AP), portal venous phase, DP, and HBP scans were performed 20–30 s, 50–60 s, 90–120 s, and 60 min after the injection of Gd-BOPTA, respectively. HBP scans were performed 120 min after injection of the contrast agent in patients with impaired liver function. Detailed scanner and scan parameters can be found in **Supplementary Table S1**.

### MR imaging analysis

MR imaging analysis was performed by two radiologists (with more than 10 years of abdominal imaging experience) who were blinded to the clinical and laboratory information. If their opinions were not consistent, a consensus decision was made after discussion. Two radiologists independently evaluated 10 imaging features defined in Liver Imaging Reporting and Data System (LI-RADS) v2018 (21): (a) tumor size, the longest axis diameter measured on HBP images; (b) radiological capsule enhancement; (c) restricted diffusion; (d) non-rim arterial phase hyperenhancement (APHE); (e) rim APHE; (f) non-peripheral washout; and (g) hepatobiliary phase hypointensity. The definition of LI-RADS features can be found in **Supplementary Table S2**.

Non-LI-RADS imaging features comprised (a) tumor number (b) shape (c) non-smooth margin (15); (d) enhancement pattern; (e) arterial peritumoral enhancement (17, 22); (f) peritumoral hypointensity on HBP (23, 24). The definition of Non-LI-RADS imaging features can be found in **Supplementary Table S3**.

### Model development, validation, and evaluation

Multivariate logistic regression (forward LR) was used to identify independent predictors of MVI and the HBP model was established. Four-fold cross validation (123 patients in the training set and 41 patients in test set) was used to verify the model, which was visualized by nomograms (25). The predictive performance of the model was evaluated based on discrimination, calibration, and clinical utility. The discrimination for the prediction model was quantified using the area under receiver operating characteristic (ROC) curve, sensitivity, specificity, and accuracy. The calibration curve analysis was conducted to evaluate the consistency between the MVI model prediction and the actual MVI state. Decision curve analysis (DCA) was conducted to determine the clinical utility of the model by quantifying the net benefits at different threshold probabilities. Net reclassification improvement (NRI) and Integrated discrimination improvement (IDI) were used to compare the diagnostic accuracy improvement level and overall improvement level between models.

### Statistical analysis

IBM SPSS Statistics (version 25; IBM) or R (version 3.6.0; http://www.r-project.org) were used for statistical analyses. Continuous variables conforming to the normal distribution and homogeneity of variance were represented as the means ± standard deviations. Inconsistent continuous variables were represented using the median (range) and compared with the Mann-Whitney U test. Categorical variables were compared using the χ2 test. Interobserver agreement between two radiologists were compared with the Kappa test, variables with kappa value < 0.75 were removed. In light of the large gradient variance, logarithmical conversion (log AFP grad and log PIVKA-II grad) was performed and used for analysis. The radiological, clinical, and pathological factors with P<0.1 in the univariate logistic regression analysis were included in multivariate logistic regression analysis (forward LR) to establish the model.

## Results

### Clinicoradiological characteristics for predicting MVI

Among the 164 patients (138 men; 55.13 ± 10.52 years), only 55 patients suffered from MVI, and 109 patients had no MVI. The comparison of Clinicoradiological characteristics is shown in **Table 1**. Using comparative analysis of the clinicopathological parameters of the MVI (n = 55) and non-MVI (n = 109) groups, it was found that the AFP_lg10 (P=0.002) and CHE (P=0.003) levels in the MVI group were higher than those in the non-MVI group.

Among the MRI features, non-smooth margin (58.2% vs. 40.4%, P=0.032), absent radiological capsule (32.1% vs. 17.4%, P=0.029), incomplete or absent radiological capsule enhancement (78.2% vs. 52.3%, P=0.002), atypical enhancement pattern (54.5% vs. 25.7%, P<0.001), rim APHE (40.0% vs. 17.4%, P=0.002), arterial peritumoral enhancement (34.5% vs. 17.4%, P=0.016), HBP hypointensity (78.2% vs. 49.5%, P=0.001), and peritumoral hypointensity on HBP (56.4% vs. 12.8%, P<0.001) had a higher probability in the MVI group than in the non-MVI group.

### Univariate and multivariate analysis factors predictive of MVI

**Figure 2:**
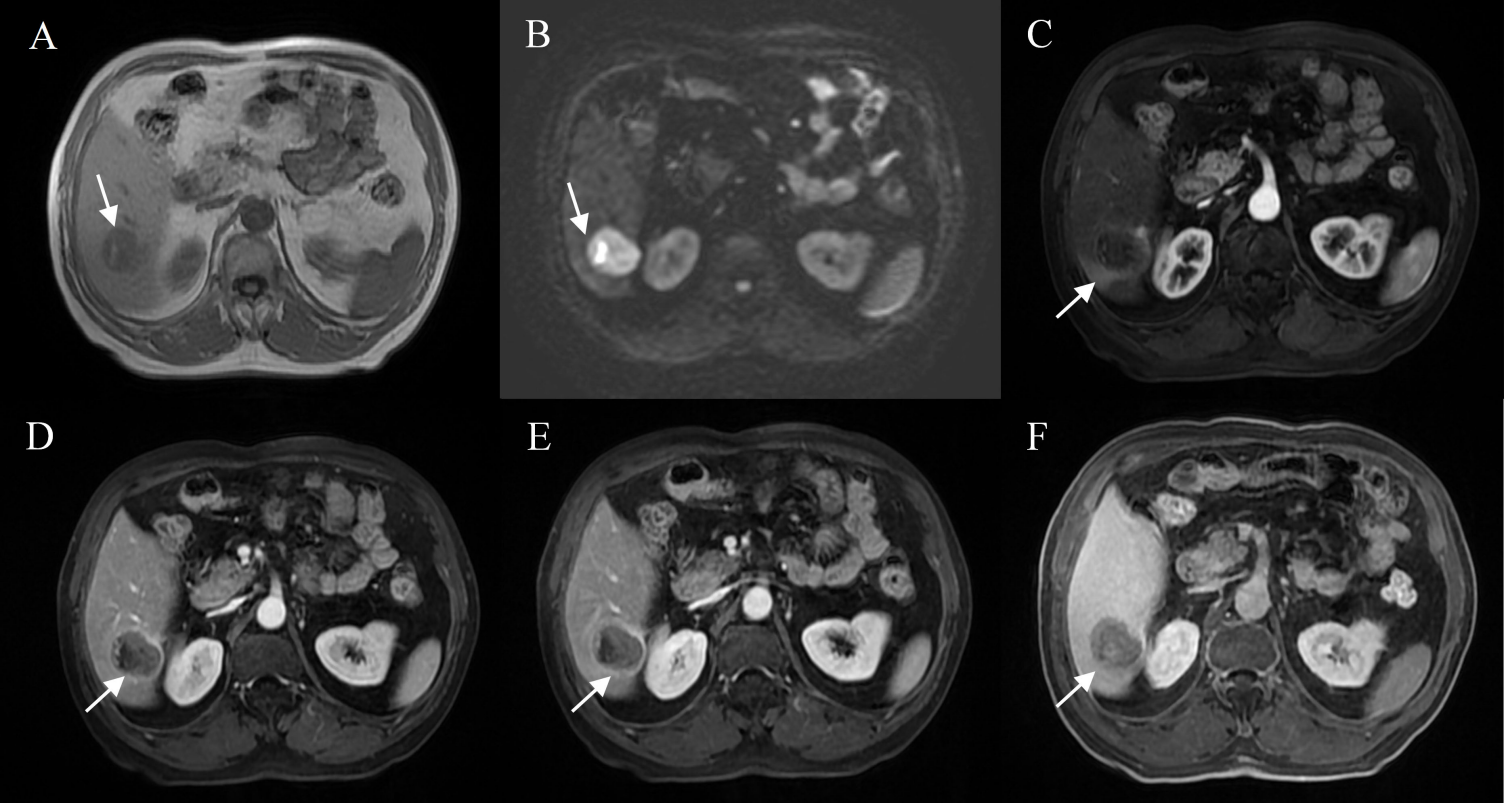
Representative images of MVI positive cases: A 71-year-old male with elevated AFP, Gd- BOPTA MRI detected a solid lesion in hepatic segment VI **(A)**, restricted diffusion **(B)**, atypical enhancement pattern without “wash-in” **(C–E)**, with the architectures of peritumoral enhancement on arterial phase images **(C)**, incomplete capsule enhancement on portal venous phase and transitional phase images **(D, E)**, and peritumoral hypointensity on hepatobiliary phase **(F)**.

**Figure 3:**
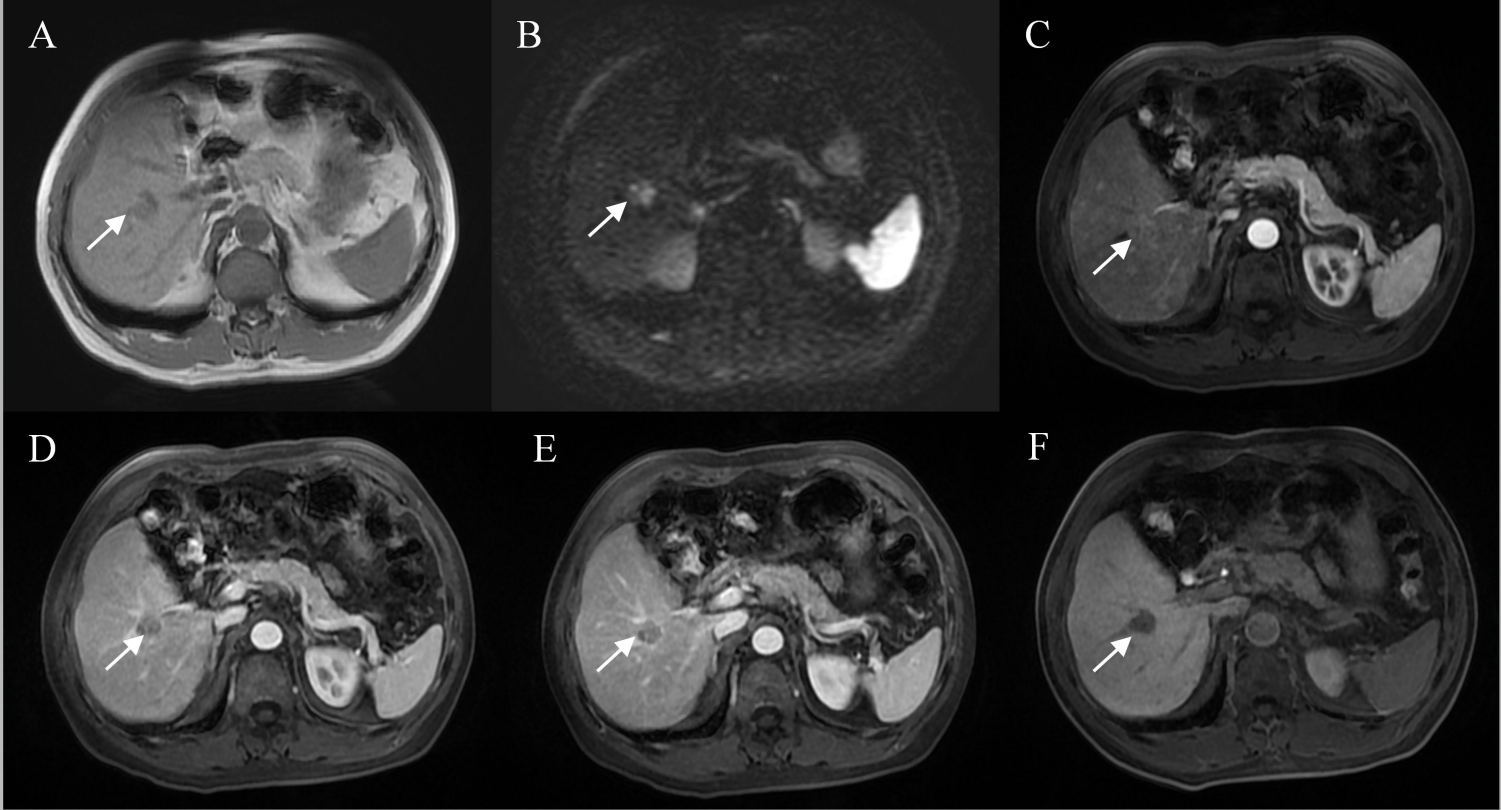
Representative images of MVI positive cases: A 60-year-old male with elevated AFP, Gd-BOPTA MRI detected a solid lesion in hepatic segment V **(A)**, restricted diffusion **(B)**, atypical enhancement pattern without “wash-in” **(C–E)**, incomplete capsule enhancement on portal venous phase and transitional phase images **(D, E)**, and homogeneous HBP hypointensity **(F)**.

**Figure 4:**
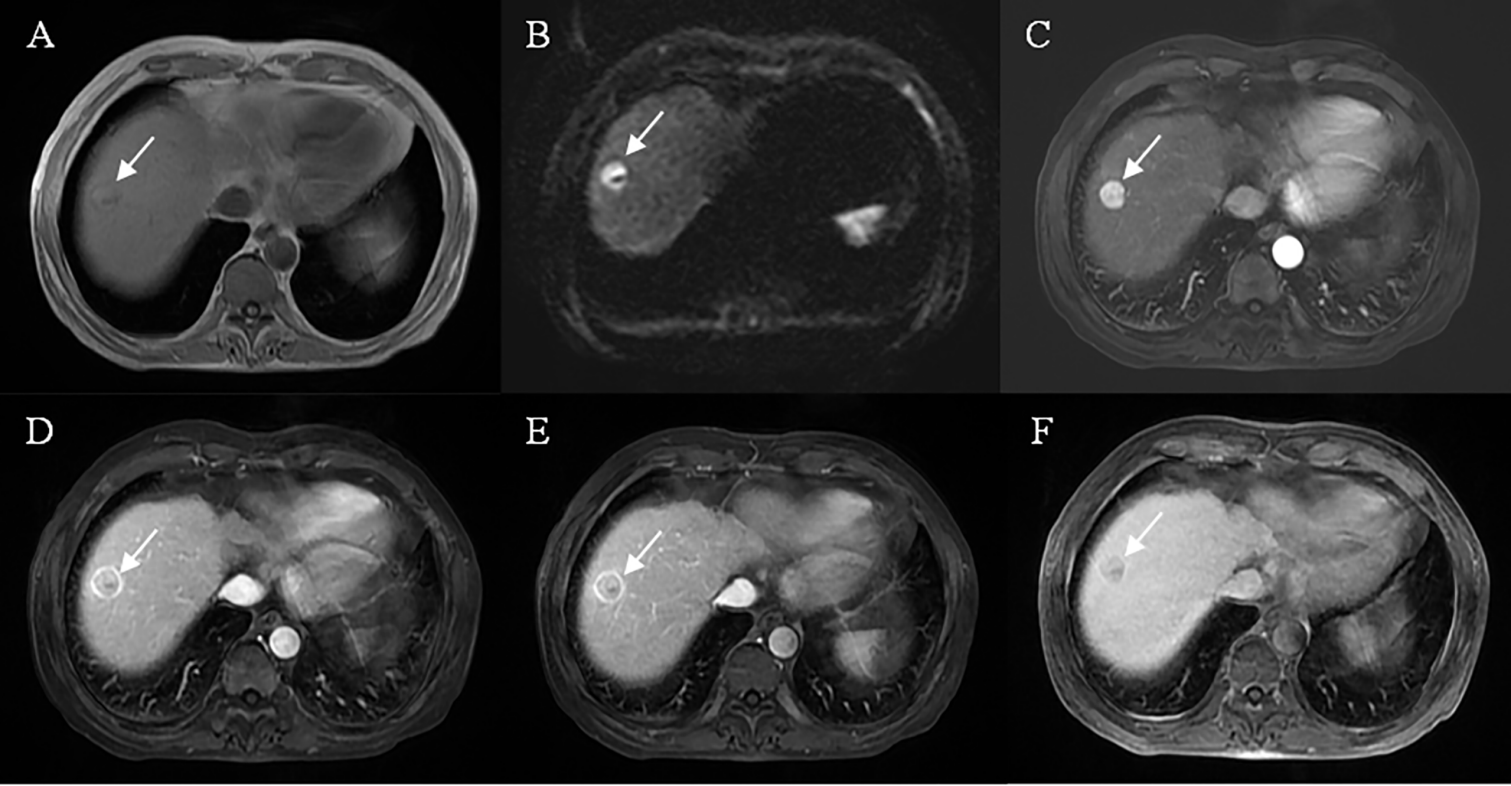
Representative images of MVI negative case: A 61-year-old male, the lesion located at hepatic segment VIII **(A)**, restricted diffusion **(B)**, but showed a well-circumscribed smooth tumor edge and complete capsule enhancement **(D, E)**, typical enhancement pattern with “wash-in” and “wash-out” **(C–E)**, without peritumoral enhancement on arterial phase images **(C)** and mild HBP hypointensity **(F)**.

A total of 12 features were related to MVI at a test level of P<0.1 in univariate analysis (**Table 2**). All of the above 12 variables were included in multivariate logistic regression analysis (forward LR), which determined that elevated AFP_lg10 (hazard ratio (HR) 1.849, 95% confidence interval (CI): 1.193, 2.867, P=0.006), atypical enhancement pattern (HR 3.441, 95% CI: 1.523, 7.772, P=0.003), peritumoral hypointensity on HBP (HR 7.822, 95% CI: 3.317, 18.445, P<0.001), and homogeneous HBP hypointensity (HR 3.258, 95% CI: 1.381, 7.687, P=0.007) were independent risk factors of MVI. Therefore, the above four risk factors constituted the HBP model (**Table 3**). The representative images of MVI cases are displayed in **Figures 2**, **3**, and non-MVI cases are displayed in **Figure 4**.

**Table 2:** Univariate analysis factors predictive of MVI.

Variable	HR (95%CI)	P Value
AFP_lg10 (ng/L)	1.784 (1.233,2.581)	0.002
Atypical enhancement pattern	3.471 (1.754,6.872)	<0.001
Arterial peritumoral enhancement	2.500 (1.188,5.262)	0.016
Radiological capsule enhancement	3.269 (1.556,6.866)	0.002
Peritumoral hypointensity on HBP	8.765 (4.043,19.003)	<0.001
Hepatobiliary phase hypointensity	3.650 (1.738,7.664)	0.001
Margin	2.055 (1.064,3.970)	0.032
Radiological capsule	2.304 (1.089,4.877)	0.029
Rim APHE	3.158 (1.519,6.566)	0.002
Non-peripheral “washout”	3.310 (1.677,6.533)	0.001
ALB (g/L)	1.049 (0.988,1.102)	0.058
CHE (U/L)	1.000 (1.000,1.000)	0.003

P Value is the p value of univariate Logistic regression analysis; HR, Hazard Ratio; Abbreviations can be found in the notes of **Table 1**.

**Table 3:** Multivariate analysis factors predictive of MVI.

	HBP model		
Variable	β	HR (95%CI)	P Value
AFP_lg10 (ng/L)	0.615	1.849 (1.193,2.867)	0.006
Atypical enhancement pattern	1.236	3.441 (1.523,7.772)	0.003
Peritumoral hypointensity on HBP	2.057	7.822 (3.317,18.445)	<0.001
Hepatobiliary phase hypointensity	1.181	3.258 (1.381,7.687)	0.007

P Value is the p value of univariate Logistic regression analysis; HR, Hazard Ratio; Abbreviations can be found in the notes of **Table 1**.

### Model development, validation and comparison

**Figure 5:**
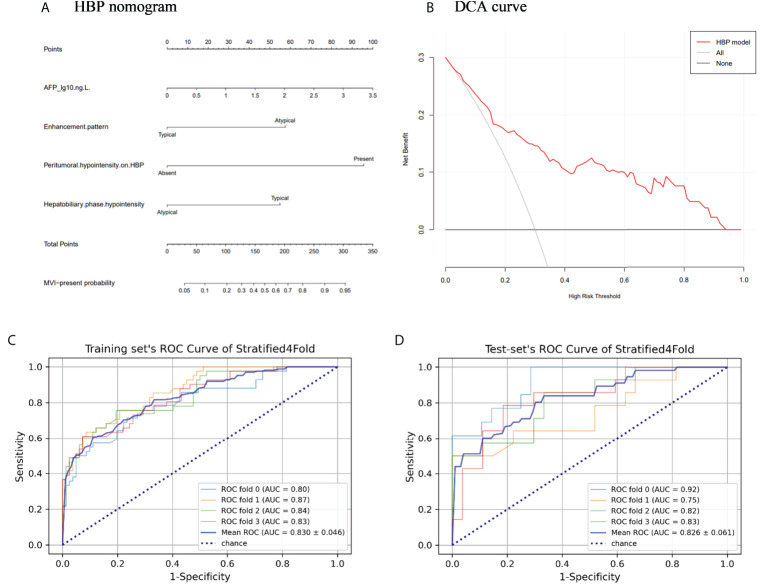
The nomogram and receiver operating characteristic curves for predicting MVI: **(A)** HBP model visualized by nomograms; **(B)** Decision curve analysis (DCA) of HBP model; **(C)** Training set’s receiver operating characteristic (ROC) curves of stratified 4-fold; **(D)** Test set’s ROC curves of stratified 4-fold.

The above four risk factors constituted the HBP model (**Table 3**), and a nomogram established based on the HBP model for predicting MVI in HCC is shown in **Figure 5A**. Four-fold cross validation was used to verify the model, the results showed that the mean area under the curve (AUC), sensitivity, specificity, and accuracy values for the HBP model were as follows: 0.830 (95% CI: 0.784, 0.876), 0.71, 0.78, 0.81 in training set; 0.826(95% CI:0.765, 0.887), 0.8, 0.7, 0.79 in test set (**Figures 5C, D**; **Table 4**). It was further evaluated using calibration curves (**Supplementary Figure S1**), which showed that the predicted MVI probability from the HBP nomogram is consistent with the estimated value of the actual MVI probability. The DCA curve for the HBP model showed that the model obtained a good net clinical benefit (**Figure 5B**). To further demonstrated the HBP model has an excellent prediction efficiency, we also established a no-HBP model after removing HBP imaging features, which constituted with elevated AFP_lg10, atypical enhancement pattern, arterial peritumoral enhancement, and capsule enhancement. Comparison between models with or without HBP imaging features determined that the NRI was 0.182 (95% CI: 0.069, 0.295, P=0.002) (**Supplementary Figure S2**), while the IDI was 0.098 (95% CI: 0.044, 0.151, P<0.001), indicating that the prediction efficiency of the HBP model is significantly improved. Therefore, the HBP model has stable and excellent prediction performance.

**Table 4:** Predictive performance for the HBP model.

	HBP model	
	Training set	Test set
Mean AUC (95%CI)	0.830 (0.784, 0.876)	0.826 (0.765, 0.887)
Sensitivity	0.71	0.8
Specificity	0.78	0.7
Accuracy	0.81	0.79

AUC, the area under the mean receiver operating characteristic curve.

## Discussion

For clinicians, MVI is essential for assessing patient prognosis and implementing appropriate treatment strategies, which has an important impact on patient survival (26, 27). The study results demonstrated that elevated AFP levels, atypical enhancement pattern, HBP hypointensity, and peritumoral hypointensity on HBP were independent risk factors for MVI. The above four risk factors constituted the HBP model. By verifying and evaluating the HBP model, the results showed that the HBP model has stable and excellent prediction performance. By comparing between models with or without HBP imaging features, the results showed that HBP model improved the prediction efficiency. Therefore, a preoperative HBP model, mainly based on HBP imaging features of Gd-BOPTA-enhanced MRI, was able to excellently predict the MVI for HCC size of ≤ 5cm. Previous study demonstrated that enlarged surgical margin (usually over 1cm) could reduce postoperative tumor recurrence rates in MVI-positive patients with HCC (8). The results of our study may help clinicians preoperatively assess the risk of MVI in HCC patients to provide preoperative guidance for clinicians to optimize treatment options.

In the present study, peritumoral hypointensity on HBP was a significant independent risk factor for predicting MVI in the HBP model, which was consistent with other research (7, 19, 20). Peritumoral hypointensity on HBP might be a result of impaired functions of peritumoral hepatocyte organic anion-transporting polypeptide transporters due to perfusion alterations in the hepatocytes around the HCC (28). It may be caused by impaired hepatocyte function (29) or Kupffer cell damage in neoplastic arterial portal shunts, as portal vein branches are blocked by cancer embolus (30). HBP hypointensity is a prominent imaging feature of HCC based on Gd-BOPTA-enhanced MRI, indicating a lack of functional hepatocytes in the tumor (31). In our study, compared with MVI-negative cases, the hepatobiliary phase was more likely to show homogeneous hypointensity rather than mild hypointensity in MVI-positive cases.

Atypical enhancement pattern was also an independent predictor of MVI, but it has been rarely reported in previous studies. HCC is unique since a non-invasive diagnosis can be achieved *via* imaging features when specific clinical criteria and imaging characteristics are met (32). However, HCC is a highly heterogeneous neoplasm (33). The atypical enhancement pattern may be attributed to different pathological subtypes (34). Atypical HCC subtypes vary widely in morphology, which can be attributed to specific histological and molecular features and may lead to atypical imaging features (35). Previous studies have shown that clear cell HCC lacks the tendency to hyperenhancement in the arterial phase and classic enhancement pattern when the proportion of clear cells was 100% (34, 35); the most common enhancement patterns in scirrhous HCC are peripheral, rim-like enhancement and late-phase progressive central enhancement (34, 35). In addition, Elevated AFP level has been reported to be independent predictors of MVI, which is in agreement with the present results (7, 15).

Arterial peritumoral enhancement and incomplete or absent capsule enhancement have been reported as independent risk factors for MVI (5, 6). However, in the present study, these imaging features were significantly susceptible to MVI but were not independent risk factors for MVI. A variety of studies have suggested that arterial peritumoral enhancement may be a result of compensatory arterial hyperperfusion leading to a reduced portal blood flow, which possibly results in occlusion of the tiny portal vein branches *via* microtumor thrombus formation around the tumor (35, 36). Some studies showed that intact capsules may protect against the dissemination and progression of HCC (33, 37). However, if cancerous cells breach the capsule, the image will show an incomplete or absent capsule enhancement and infiltrative border (non-smooth margin) (37). The present data showed that intact capsules are more common in patients without MVI.

The strengths of this study include the use of the hepatobiliary-specific contrast agent Gd-BOPTA, which obtained HBP imaging features. Previous studies have reported some models for predicting MVI using gadoxetate disodium-enhanced MRI (16–18), but no previous literature has reported the use of gadolinate-enhanced MRI to build a model to predict MVI for HCC. Furthermore, the present study focused on the HCC size within 5 cm, which reduced the confounding effect from tumor size. At present, the radical treatment rate of patients with HCC size of ≤5 cm has been significantly improved, which has become a practical problem that urgently needs to be solved in hepatic surgery.

The study has several limitations. First, the retrospective single-center nature of the study might have introduced selection biases. Second, because few hospitals are using Gd-BOPTA hepatobiliary-specific contrast agent, external validation was not conducted. Third, the enrolled cases were mainly concentrated in tumors with sizes of ≤5 cm, resulting in a slightly lower frequency of MVI in the study population. Therefore, the reliability and robustness of these findings should be validated in future studies with larger HCCs.

In conclusion, the HBP imaging features of Gd-BOPTA-enhanced MRI play an important role in predicting MVI for HCC. A preoperative HBP model, mainly based on HBP imaging features of Gd-BOPTA-enhanced MRI, was able to successfully predict the MVI for HCC size of ≤ 5cm. The model may help clinicians preoperatively assess the risk of MVI in HCC patients so as to guide clinicians to optimize treatment options.

## Data availability statement

The original contributions presented in the study are included in the article/**Supplementary Material**. Further inquiries can be directed to the corresponding authors.

## Ethics statement

The studies involving human participants were reviewed and approved by The Eastern Hepatobiliary Surgery Hospital, the Third Affiliated Hospital of Shanghai Naval Military Medical University, China. Written informed consent for participation was not required for this study in accordance with the national legislation and the institutional requirements.

## Author contributions

SZ conceived the project and designed the study. LH, YF, YL, JZ, and YW performed the data extraction and collection. WL and SZ performed the data analysis. SZ, WL, and NJ wrote and revised the manuscript. All authors contributed to the article and approved the submitted version.

## Funding

This research was supported by the grants from the Clinical Research Plan of SHDC (grant number: SHDC2020CR1029B-002).

## Conflict of interest

The authors declare that the research was conducted in the absence of any commercial or financial relationships that could be construed as a potential conflict of interest.

## Publisher’s note

All claims expressed in this article are solely those of the authors and do not necessarily represent those of their affiliated organizations, or those of the publisher, the editors and the reviewers. Any product that may be evaluated in this article, or claim that may be made by its manufacturer, is not guaranteed or endorsed by the publisher.

## References

[1] Yang JD Hainaut P Gores GJ Amadou A Plymoth A Roberts LR. A global view of hepatocellular carcinoma: trends, risk, prevention and management. Nat Rev Gastroenterol Hepatol (2019) 16(10):589–604. doi: 10.1038/s41575-019-0186-y 31439937 PMC6813818

[2] Fujiwara N Liu PH Athuluri-Divakar SK Zhu S Hoshida Y. Risk factors of hepatocellular carcinoma for precision personalized care. In: Y Hoshida, editor. Hepatocellular carcinoma: Translational precision medicine approaches. Cham (CH): Humana Press (2019). p. 3–25. doi: 10.1007/978-3-030-21540-8_1 32078275

[3] Iyer RV Maguire O Kim M Curtin LI Sexton S Fisher DT. Dose-dependent sorafenib-induced immunosuppression is associated with aberrant NFAT activation and expression of PD-1 in T cells. Cancers (Basel) (2019) 11(5):681. doi: 10.3390/cancers11050681 PMC6562672 31100868

[4] Chen D Wang Y Lu R Jiang X Chen X Meng N. E3 ligase ZFP91 inhibits hepatocellular carcinoma metabolism reprogramming by regulating PKM splicing. Theranostics (2020) 10(19):8558–72. doi: 10.7150/thno.44873 PMC7392027 32754263

[5] Hong SB Choi SH Kim SY Shim JH Lee SS Byun JH. MRI Features for predicting microvascular invasion of hepatocellular carcinoma: A systematic review and meta-analysis. Liver Cancer (2021) 10(2):94–106. doi: 10.1159/000513704 33981625 PMC8077694

[6] He M Zhang P Ma X He B Fang C Jia F. Radiomic feature-based predictive model for microvascular invasion in patients with hepatocellular carcinoma. Front Oncol (2020) 10:574228. doi: 10.3389/fonc.2020.574228 33251138 PMC7674833

[7] Zhang XP Wang K Wei XB Li LQ Sun HC Wen TF. An Eastern hepatobiliary surgery hospital microvascular invasion scoring system in predicting prognosis of patients with hepatocellular carcinoma and microvascular invasion after R0 liver resection: A Large-scale, multicenter study. Oncologist (2019) 24(12):e1476–88. doi: 10.1634/theoncologist.2018-0868 PMC6975940 31138726

[8] Orcutt ST Anaya DA. Liver resection and surgical strategies for management of primary liver cancer. Cancer Control (2018) 25(1):1073274817744621. doi: 10.1177/1073274817744621 29327594 PMC5933574

[9] Wang YY Wang LJ Xu D Liu M Wang HW Wang K. Postoperative adjuvant transcatheter arterial chemoembolization should be considered selectively in patients who have hepatocellular carcinoma with microvascular invasion. HPB (Oxford) (2019) 21(4):425–33. doi: 10.1016/j.hpb.2018.08.001 30249510

[10] Zhang J Huang S Xu Y Wu J. Diagnostic accuracy of artificial intelligence based on imaging data for preoperative prediction of microvascular invasion in hepatocellular carcinoma: A systematic review and meta-analysis. Front Oncol (2022) 12:763842. doi: 10.3389/fonc.2022.763842 35280776 PMC8907853

[11] Lee S Kim SH Lee JE Sinn DH Park CK. Preoperative gadoxetic acid-enhanced MRI for predicting microvascular invasion in patients with single hepatocellular carcinoma. J Hepatol (2017) 67(3):526–34. doi: 10.1016/j.jhep.2017.04.024 28483680

[12] Li Y Chen J Weng S Yan C Ye R Zhu Y. Hepatobiliary phase hypointensity on gadobenate dimeglumine-enhanced magnetic resonance imaging may improve the diagnosis of hepatocellular carcinoma. Ann Transl Med (2021) 9(1):55. doi: 10.21037/atm.2020.02.38 33553348 PMC7859813

[13] Liu C Sun Y Yang Y Feng Y Xie X Qi L. Gadobenate dimeglumine-enhanced biliary imaging from the hepatobiliary phase can predict progression in patients with liver cirrhosis. Eur Radiol (2021) 31(8):5840–50. doi: 10.1007/s00330-021-07702-6 33533990

[14] Bonnaventure P Cusin F Pastor CM. Hepatocyte concentrations of imaging compounds associated with transporter inhibition: Evidence in perfused rat livers. Drug Metab Dispos (2019) 47(4):412–8. doi: 10.1124/dmd.118.084624 30674615

[15] Zhang L Yu X Wei W Pan X Lu L Xia J. Prediction of HCC microvascular invasion with gadobenate-enhanced MRI: correlation with pathology. Eur Radiol (2020) 30(10):5327–36. doi: 10.1007/s00330-020-06895-6 32367417

[16] Wei H Jiang H Liu X Qin Y Zheng T Liu S. Can LI-RADS imaging features at gadoxetic acid-enhanced MRI predict aggressive features on pathology of single hepatocellular carcinoma? Eur J Radiol (2020) 132:109312. doi: 10.1016/j.ejrad.2020.109312 33022551

[17] Chen ZH Zhang XP Wang H Chai ZT Sun JX Guo WX. Effect of microvascular invasion on the postoperative long-term prognosis of solitary small HCC: a systematic review and meta-analysis. HPB (Oxford) (2019) 21(8):935–44. doi: 10.1016/j.hpb.2019.02.003 30871805

[18] Ahn SJ Kim JH Park SJ Kim ST Han JK. Hepatocellular carcinoma: preoperative gadoxetic acid-enhanced MR imaging can predict early recurrence after curative resection using image features and texture analysis. Abdom Radiol (NY) (2019) 44(2):539–48. doi: 10.1007/s00261-018-1768-9 30229421

[19] Cong WM Bu H Chen J Dong H Zhu YY Feng LH. Guideline committee. practice guidelines for the pathological diagnosis of primary liver cancer: 2015 update. World J Gastroenterol (2016) 22(42):9279–87. doi: 10.3748/wjg.v22.i42.9279 PMC5107692 27895416

[20] Cai Y Fu Y Liu C Wang X You P Li X. Stathmin 1 is a biomarker for diagnosis of microvascular invasion to predict prognosis of early hepatocellular carcinoma. Cell Death Dis (2022) 13(2):176. doi: 10.1038/s41419-022-04625-y 35210426 PMC8873260

[21] Rimola J Sapena V Brancatelli G Darnell A Forzenigo L Mähringer-Kunz A. Reliability of extracellular contrast vs. gadoxetic acid in assessing small liver lesions using LI-RADS v.2018 and EASL criteria. Hepatology (2022). doi: 10.1002/hep.32494 35349760

[22] Renzulli M Brocchi S Cucchetti A Mazzotti F Mosconi C Sportoletti C. Can current preoperative imaging be used to detect microvascular invasion of hepatocellular carcinoma? Radiology (2016) 279(2):432–42. doi: 10.1148/radiol.2015150998 26653683

[23] Tsochatzis EA Bosch J Burroughs AK. Liver cirrhosis. Lancet (2014) 383(9930):1749–61. doi: 10.1016/s0140-6736(14)60121-5 24480518

[24] Liang W Zhang L Jiang G Wang Q Liu L Liu D. Development and validation of a nomogram for predicting survival in patients with resected non-small-cell lung cancer. J Clin Oncol (2015) 33(8):861–9. doi: 10.1200/JCO.2014.56.6661 25624438

[25] Vickers AJ Cronin AM Elkin EB Gonen M. Extensions to decision curve analysis, a novel method for evaluating diagnostic tests, prediction models and molecular markers. BMC Med Inform Decis Mak (2008) 8:53. doi: 10.1186/1472-6947-8-53 19036144 PMC2611975

[26] Ma X Wei J Gu D Zhu Y Feng B Liang M. Preoperative radiomics nomogram for microvascular invasion prediction in hepatocellular carcinoma using contrast-enhanced CT. Eur Radiol (2019) 29(7):3595–605. doi: 10.1007/s00330-018-5985-y 30770969

[27] Li H Li T Hu J Liu J. A nomogram to predict microvascular invasion in early hepatocellular carcinoma. J Cancer Res Ther (2021) 17(3):652–7. doi: 10.4103/jcrt.JCRT_1714_20 34269295

[28] Chong H Zhou P Yang C Zeng M. An excellent nomogram predicts microvascular invasion that cannot independently stratify outcomes of small hepatocellular carcinoma. Ann Transl Med (2021) 9(9):757. doi: 10.21037/atm-20-7952 34268370 PMC8246205

[29] Jiang H Wei J Fu F Wei H Qin Y Duan T. Predicting microvascular invasion in hepatocellular carcinoma: A dual-institution study on gadoxetate disodium-enhanced MRI. Liver Int (2022) 42(5):1158–72. doi: 10.1111/liv.15231 PMC9314889 35243749

[30] Wang LL Li JF Lei JQ Guo SL Li JK Xu YS. The value of the signal intensity of peritumoral tissue on gd-EOB-DTPA dynamic enhanced MRI in assessment of microvascular invasion and pathological grade of hepatocellular carcinoma. Med (Baltimore) (2021) 100(20):e25804. doi: 10.1097/MD.0000000000025804 PMC8136999 34011043

[31] Shin SK Kim YS Shim YS Choi SJ Park SH Jung DH. Peritumoral decreased uptake area of gadoxetic acid enhanced magnetic resonance imaging and tumor recurrence after surgical resection in hepatocellular carcinoma: A STROBE-compliant article. Med (Baltimore) (2017) 96(33):e7761. doi: 10.1097/MD.0000000000007761 PMC5571690 28816953

[32] Wei Y Huang Z Tang H Deng L Yuan Y Li J. IVIM improves preoperative assessment of microvascular invasion in HCC. Eur Radiol (2019) 29(10):5403–14. doi: 10.1007/s00330-019-06088-w 30877465

[33] Karadag Soylu N. Update on hepatocellular carcinoma: a brief review from pathologist standpoint. J Gastrointest Cancer (2020) 51(4):1176–86. doi: 10.1007/s12029-020-00499-5 32844348

[34] Bello HR Mahdi ZK Lui SK Nandwana SB Harri PA Davarpanah AH. Hepatocellular carcinoma with atypical imaging features: Review of the morphologic hepatocellular carcinoma subtypes with radiology-pathology correlation. J Magn Reson Imaging (2022) 55(3):681–97. doi: 10.1002/jmri.27553 33682266

[35] Yoon JK Choi JY Rhee H Park YN. MRI Features of histologic subtypes of hepatocellular carcinoma: correlation with histologic, genetic, and molecular biologic classification. Eur Radiol (2022) 32(8):5119–33. doi: 10.1007/s00330-022-08643-4 35258675

[36] Choi JY Lee JM Sirlin CB. CT and MR imaging diagnosis and staging of hepatocellular carcinoma: part II. extracellular agents, hepatobiliary agents, and ancillary imaging features. Radiology (2014) 273(1):30–50. doi: 10.1148/radiol.14132362 25247563 PMC4263770

[37] Cho ES Choi JY. MRI Features of hepatocellular carcinoma related to biologic behavior. Korean J Radiol (2015) 16(3):449–64. doi: 10.3348/kjr.2015.16.3.449 PMC4435980 25995679

