# Supplementary material for: A preoperative model based on gadobenate-enhanced MRI for predicting microvascular invasion in hepatocellular carcinomas (≤ 5 cm)
Source: Front Oncol. 2022 Aug 30;12:992301. doi: 10.3389/fonc.2022.992301 (PMC9470230; doi:10.3389/fonc.2022.992301)
Supplement: Supplementary file 1 [file DataSheet_1.docx]

Supplementary Material

**Supplementary Table S1.** **Detailed scanner and scan parameters.**

| **Sequences** | **Image plane** | **TR/TE (msec)** | **FOV (mm)** | **Flip angle** | **Thickness (mm)** | **Matrix** | **Scanning order** |
| --- | --- | --- | --- | --- | --- | --- | --- |
| FIESTA | C | 3.5/1.5 | 420 × 420 | 60 | 6 | 160 × 224 | 1 |
| T1WI | A | 190/4.3(2) | 420 × 420 | 80 | 6 | 256 × 160 | 3 |
| T2WI | A | 6667/85 | 420 × 420 | 160 | 6 | 320 × 224 | 4 |
| LAVA | A | 3.7/1.7 | 420 × 420 | 15 | 2.5 | 256 × 192 | 5 |
| 2D MRCP | Oblique | 4000/847 | 320 × 320 | 160 | 50 | 320 × 256 | 6 |
| HBP | A | 3.7/1.7 | 420 × 420 | 15 | 2.5 | 256 × 192 | 7 |
| HBP | C | 3.7/1.7 | 400 × 400 | 15 | 2.5 | 256 × 192 | 8 |

**Supplementary Table S2. The definition of LI-RADS features.**

| LI-RADS features | Definition |
| --- | --- |
| MRI Tumor diameter(cm） | Largest outer-edge-to-outer-edge dimension of an observation: Include “capsule” in measurement；Pick phase, sequence, plane in which margins are clearest；Do not measure in arterial phase or DWI if margins are clearly visible on different phase (size may be overestimated in arterial phase due to summation with periobservation enhancement and is not measured reliably on DWI due to anatomic distortion). |
| Radiological capsule enhancement | Enhancing “capsule”: Smooth, uniform, sharp border around most(incomplete) or all(complete) of an observation, unequivocally thicker or more conspicuous than fibrotic tissue around background nodules, and visible as enhancing rim in PVP, DP, or TP; Absent (Non-enhancing “capsule”): Capsule appearance not visible as an enhancing rim. |
| Restricted diffusion | Intensity on DWI, not attributable solely to T2 shine-through, unequivocally higher than liver and/or ADC unequivocally lower than liver. |
| Non-rim APHE | Nonrim-like enhancement in arterial phase unequivocally greater in whole or in part than liver. Enhancing part must be higher in attenuation or intensity than liver in arterial phase. Contrast with rim APHE. |
| Rim APHE | Spatially defined subtype of APHE in which arterial phase enhancement is most pronounced in observation periphery. |
| Non peripheral washout | Nonperipheral visually assessed temporal reduction in enhancement in whole or in part relative to composite liver tissue from earlier to later phase resulting in hypoenhancement in the extracellular phase: portal venous or delayed phase if ECA or gadobenate is given; portal venous phase if gadoxetate is given. |
| Hepatobiliary phase hypointensity | Intensity in the hepatobiliary phase unequivocally less, in whole or in part, than liver. Typical HBP sign is homogeneous hypointensity, and atypical HBP sign is not homogeneous hypointensity. |

**Supplementary Table S3 The definition of Non-LI-RADS imaging features**

| Non-LI-RADS features | Definition |
| --- | --- |
| Tumor number | Only one lesion was solitary, and two or more lesions were multiple. |
| Shape | Round or oval were defined as regular, while others were defined as irregular, including lobulated, star awn, and needle-like. |
| Margin | Nodular tumors with smooth boundary were smooth margin, non-nodular tumors with irregular contour and budding into the surrounding liver parenchyma were non-smooth margin. |
| Enhancement pattern | Typical enhancement meets the "wash in and wash out" enhancement, and the rest were typical; |
| Arterial peritumoral enhancement | Defined as the enhancement outside the tumor boundary in the late stage of AP or early stage of portal phase and extensive contact with the tumor edge, which becomes isointense during the DP. |
| Peritumoral hypointensity on HBP | Presenting as hypointense areas of liver parenchyma around the tumor boundary in crescent, wedge, or flame shapes on HBP images. |


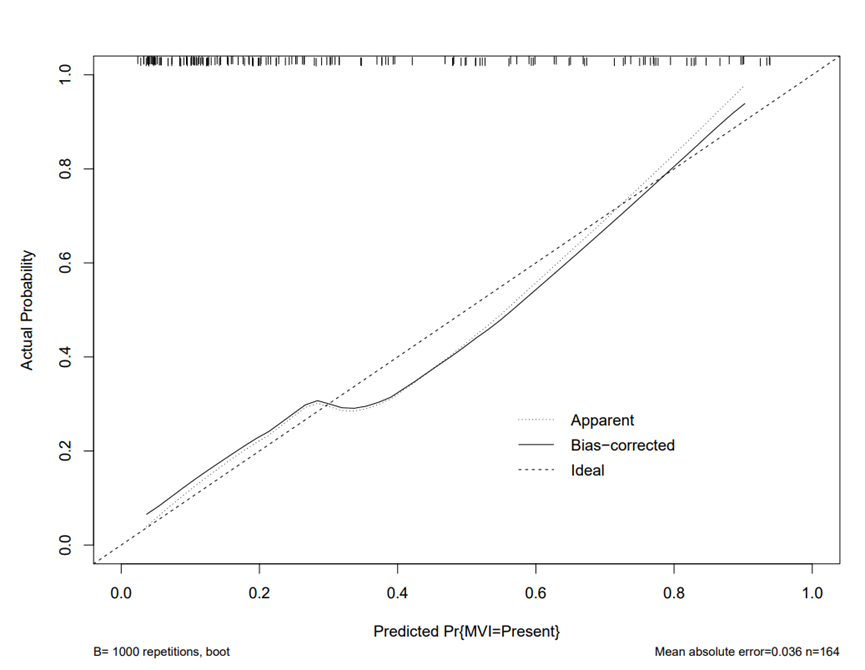
**Supplementary Figure 1. The calibration curves of the HBP model**

**
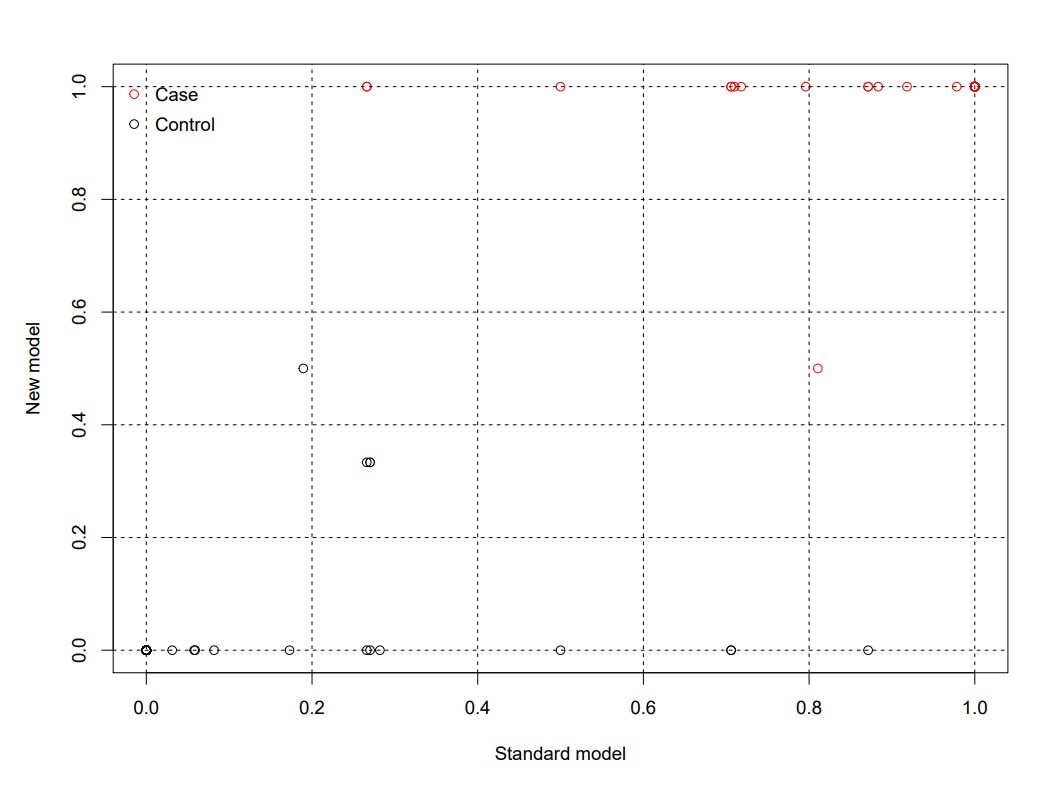
Supplementary Figure 2. The NRI for the HBP model vs. no-HBP model**
